# Invasive mycoses in patients with connective tissue disease from Southern China: clinical features and associated factors

**DOI:** 10.1186/s13075-019-1851-9

**Published:** 2019-03-11

**Authors:** Minxi Lao, Zhongping Zhan, Fan Su, Hao Li, Zheng Yang, Haihong Chen, Liuqin Liang, Dongying Chen

**Affiliations:** 1 Department of Rheumatology & Geriatrics, The First Affiliated Hospital of Sun Yat-sen University, Guangzhou, China; 2 Department of Rheumatology, The First Affiliated Hospital of Sun Yat-sen University, 58 Zhongshan 2nd Road, Guangzhou, 510080 China; 3 Department of Pathology, The Seventh Affiliated Hospital of Sun Yat-sen University, Shenzhen, China; 4 Department of Respirology, The First Affiliated Hospital of Sun Yat-sen University, Guangzhou, China

**Keywords:** Connective tissue disease, Invasive mycosis, Lymphopenia, Glucocorticoid, Co-infection

## Abstract

**Background:**

A retrospective study was performed to investigate the clinical features and associated factors of invasive mycoses (IM) in patients with connective tissue disease (CTD) from Southern China.

**Methods:**

Demographic and clinical data were recorded. Associated factors were analyzed by logistic regression analysis.

**Results:**

A total of 6911 patients with CTD were included. IM was diagnosed in 32 patients (incidence, 0.5%). IM was predominant in patients with ANCA-associated vasculitis (AAV) (incidence, 1.5%, 7/480). Lung was commonly involved (30/32, 93.8%). *Aspergillus* spp. (81.3%) were the leading strain. The positive rate of fungi detection in sputum culture was 69.0%. Serum galactomannan (GM) test was positive in bronchoalveolar lavage fluid (BALF) from seven (7/10, 70.0%) patients. Ten patients died (31.3%), including three with AAV (42.9%) and seven with SLE (36.8%). *Penicillium marneffei* was the most fatal (mortality, 100%). Non-survivors had higher prevalence of leukopenia (30.0% vs 4.5%, *P* = 0.04), lymphopenia (100.0% vs 59.1%, *P* = 0.02), elevated serum creatinine (70.0% vs 27.3%, *P* = 0.02), and co-infection (70.0% vs 18.2%, *P* = 0.004) than survivors. Multivariate logistic regression analysis showed that lymphopenia [odds ratio (OR) = 3.28, 95% confidence interval (CI) 1.29–8.38, *P* = 0.01] and median-to-high dose of glucocorticoid (GC) [OR = 3.40, 95% CI 1.04–11.13, *P* = 0.04] were associated with IM in patients with CTD.

**Conclusions:**

IM tended to develop in patients with AAV, resulting in high mortality. Sputum culture and GM test in BALF were effective methods to distinguish IM. Vigilance against lymphopenia, impaired kidney function, and co-infection improved the prognosis of IM.

**Electronic supplementary material:**

The online version of this article (10.1186/s13075-019-1851-9) contains supplementary material, which is available to authorized users.

## Background

Connective tissue disease (CTD) is a group of autoimmune diseases caused by immune system disorder. Despite considerable progress in immunosuppressive and supportive treatment, infection is still one of the major threats to patients with CTD [1]. A multicenter retrospective study found that 65.8% of the deaths were imputable to infection in patients with systemic lupus erythematosus (SLE) [2]. During the induction therapy of ANCA-associated vasculitis (AAV), the incidence of severe infection was estimated to be 34.7% [3]. Infection was also reported to be the leading cause of non-systemic sclerosis (SSc)-related deaths (18.2%) [4]. Opportunistic fungal infection was an independent factor with mortality in polymyositis/dermatomyositis (PM/DM) patients [5].

Invasive fungal disease (IFD) was well studied in patients with AIDS and organ transplant recipients [6, 7]. A few studies illustrated that patients with CTD were also predisposed to IFD. A systemic review included 182 studies reported that the prevalence of IFD in lupus patients ranged between 0.6 and 3.2% [8]. A total of 461 cases of IFD were included in the review, and mold infection accounted for 21.9%. Death rate increased four- to sixfold in lupus patients with IFD than those without [9, 10]. Another research showed that among 87 episodes of severe infection in patients with new diagnosis of AAV, 3 (3.4%) were caused by *Aspergillus* spp. [3]. A Chinese research exploring invasive pulmonary aspergillosis (IPA) in hospitalized patients with AAV reported that mortality of aspergillosis was as high as 57.2% [11].

Although IFD, especially invasive mycosis (IM) caused by molds, is a challenging cause of morbidity and mortality of CTD, our present understanding is mostly limited to the typical clinical manifestation and radiological presentation. Few studies were designed to focus on IM in patients with CTD. In fact, the great variations and protean manifestation of these clinical features often lead to misdiagnosis [12]. The timing of treatment initiation against IM is not fully determined.

In this study, we performed a retrospective study in patients with SLE, AAV, PM/DM, rheumatoid arthritis (RA), and SSc, aiming to identify the epidemiology and the associated factors affecting the development of IM infected by molds in patients with CTD from Southern China.

## Methods

### Study design and case definition

We performed a retrospective study on the inpatients from the First Affiliated Hospital of Sun Yat-Sen University from January 1, 2007, to December 31, 2017. The ICD-10 coding of discharged diagnoses was used to identify patients with CTD and IM (relevant ICD-10 coding in Appendix). Records were re-evaluated by a rheumatologist (Minxi Lao) and a respirologist (Haihong Chen). SLE was diagnosed according to the 1997 American College of Rheumatology classification criteria [13]. RA was diagnosed according to the 1987 ACR revised criteria for the classification of rheumatoid arthritis [14]. AAV was determined using the definitions of the Chapel Hill nomenclature [15, 16]. PM/DM was established referring to the classification criteria proposed by Bohan [17]. The diagnosis of SSc was conducted based on 1980 ARA classification criteria for systemic sclerosis [18]. IM infected by molds was determined using the EORTC/MSG 2008 Guidelines [19] and classified into three categories namely proven, probable, and possible. Diagnosis of proven IM required culture or microscopic evidence of the molds from a normally sterile site. A probable case required the presence of a host factor, mycological evidence {identification of mold species in respiratory samples [positive twice in microscopic examination and culture of sputum, or positive once in bronchoalveolar lavage fluid (BALF)], or identification of fungal antigen in blood [twice elevated serum 1,3-β-d-glucan level (G test)], or positive twice for serum galactomannan (GM) antigen)}, and clinical manifestation as well as radiographic imaging specific to the disease entity. Possible IM was a case with a host factor and clinical presentation, but no mycological evidence. In the interest of capturing cases which were most likely to be truly IM, only proven and probable cases were included in this study. Superficial infection, namely cutaneous and mucosal infection, was excluded. Patients with incomplete data were excluded. Co-infection with other agents including bacteria, virus, or superficial fungi was diagnosed according to the criteria described previously [20]. The outcome of IM was classified into two categories including improvement and progression in our research. Improvement was defined as both clinical symptoms and radiographic imaging partially resolved. Progressive disease was defined as clinical symptoms and radiographic imaging worsened. Inpatients with CTD but no history of any infection from the same period of time were chosen as controls. Cases and controls were matched 1:2 by age, gender, subtype, and the duration of CTD (from CTD onset to IM).

### Clinical variables

Demographic and clinical data were collected from medical records. Clinical characteristics of IM included symptoms and sign, sites of involvement, and medication history. CTD-associated pneumonia was established according to the Chinese expert-based consensus statement [21]. Laboratory data included routine blood tests, erythrocyte sedimentation rate (ESR), and serum C-reactive protein (CRP). Specimen culture and biopsy finding were recorded if available. Medication history included daily dose of glucocorticoid (GC) and the use of immunosuppressive agents within the 3 months prior to IM. Median-to-high dose of GC was defined as the average dose of equivalent prednisone over 0.5 mg/kg/day within the previous 3 months. The dose of GC was converted using the following equation: 1 mg of prednisone = 0.8 mg of methylprednisolone = 0.15 mg of dexamethasone.

### Statistical analysis

Statistical analysis was computed with SPSS 19.0 software (SPSS Inc., Chicago, IL, USA). The descriptive variables included mean (S.D.) or median (interquartile range, IQR) depending on the distribution of continuous variables and number (percentage) for qualitative variables. Between-group comparison was evaluated with *chi*-square test or Fisher exact test for categorical variables and Student’s *t* test for continuous variables with normal distribution. Between-group comparison was evaluated using the Mann-Whitney *U* test for continuous variables with non-normal distribution. Variables with *P* value < 0.10 from the univariate analysis were considered as candidates for the multivariate analysis. The multivariate analysis was preceded by a forward procedure to identify the factors associated with IM. Associated factors were reported with *P* values, odds ratio (OR), and 95% confidence interval (CI). *P* value < 0.05 was considered statistically significant.

## Results

### Demographic data

A total of 6911 inpatients with CTD (including 4196 patients with SLE, 728 with PM/DM, 666 with primary Sjogren’s syndrome (pSS), 530 with vasculitis, 407 with RA, 197 with SSc, 102 with spondyloarthrosis (SpA), and 85 with mixed connective tissue disease (MCTD)) were selected. IM was diagnosed in 54 patients including 29 patients with SLE, 15 patients with AAV, 5 patients with PM/DM, 4 patients with RA, and 1 patient with SSc. Sixteen patients were excluded for possible diagnosis of IM, and six patients were excluded for data incompleteness. Finally, 32 patients (included 19 patients with SLE, 7 with AAV, 3 with PM/DM, 2 with RA, and 1 with SSc) were included in our research. Female-to-male ratio was 25:7. The incidence of IM was 0.5% (32/6911). To be specific, the incidence was 1.5% (7/480) in patients with AAV, 0.5% (19/4196) in SLE, 0.5% (2/407) in RA, 0.5% (1/197) in SSc, and 0.4% (3/728) in PM/DM. Proven infection accounted for 18.8% (6/32) of the cases, and probable infection for 81.3% (26/32). All patients were of Chinese Han ethnicity. Of the 32 patients enrolled, mean age was 42.4 ± 15.8 years old (range, 14–68). Characteristics of patients with CTD are shown in Table 1. Kidney involvement occurred in 15 (78.9%) patients with SLE and in 4 (57.1%) patients with AAV. CTD-associated interstitial pneumonia was predominant in patients with AAV (42.9%), followed by PM (33.3%) and SLE (5.3%). During the 3 months prior to IM, 22 (68.8%) patients received GC treatment, among which 50.0% (11/22) received median-to-high dose of GC. Cyclophosphamide (CYC), mycophenolate mofetil (MMF), cyclosporine A (CsA), and methotrexate (MTX) were prescribed to 9 (28.1%), 3 (9.4%), 3 (9.4%), and 2 (6.3%) patients, respectively. No patients received biologic treatment.

**Table 1:** Characteristics of patients diagnosed with CTD and IM

Characteristics	SLE (*n* = 19)	AAV (*n* = 7)	PM/DM (*n* = 3)	RA (*n* = 2)	SSc (*n* = 1)
Demographic characteristics					
Sex, male to female	5:14	1:6	1:2	0:2	0:1
Age, years, mean ± SD	35.6 ± 15.3	53.1 ± 13.0	57.0 ± 4.4	46.5 ± 9.2	46.0
Duration of CTD, months, median (IQR)	4(1.0,21.0)	3(0.3,84.0)	120(61.5,180.0)	204(186.0,222.0)	108.0
Diabetes mellitus, *n* (%)	3(15.8)	1(14.3)	0(0)	0(0)	0(0)
Nephritis, *n* (%)	15(78.9)	4(57.1)	0(0)	0(0)	0(0)
Interstitial pneumonia, *n* (%)	1(5.3)	3(42.9)	1(33.3)	0(0)	1(100.0)
Laboratory data					
WBC count (× 109), mean ± SD	9.5 ± 6.7	10.7 ± 6.0	12.9 ± 5.6	8.8 ± 1.2	6.6
Lymphocyte count (× 109), mean ± SD	0.9 ± 1.0	0.8 ± 0.7	1.1 ± 0.4	1.5 ± 1.0	0.7
Hemoglobin (g/l), mean ± SD	96.1 ± 26.2	87.3 ± 27.5	116.7 ± 2.5	114.5 ± 3.5	109.0
Serum albumin (g/l), mean ± SD	28.1 ± 7.4	32.8 ± 3.5	27.0 ± 11.3	35.5 ± 4.9	32.0
Elevated serum creatinine, *n* (%)	6(31.6)	6(85.7)	0(0)	0(0)	0(0)
IgM (g/l), median (IQR)	0.7(0.3,0.9)	0.5(0.4,0.8)	1.2(1.1,1.4)	0.7(0,0.5)	0.3
IgG (g/l), median (IQR)	15.4(7.4,17.4)	13.4(12.6,20.4)	15.2(14.4,16.0)	14.8(0,11.8)	14.1
ESR (mm/h),median (IQR)	45.0(39,58.5)	63.0(60,66.0)	87.5(81.8,93.3)	49.5(43.3,55.8)	30.0
CRP (mg/l), median (IQR)	11.0(6.2,33.4)	12.3(6.3,20.1)	102.0(52.7,139.0)	65.6(34.4,96.8)	0.6
Treatment prior to IM					
Median-to-high dose of GC, *n* (%)	6(31.6)	3(42.9)	2(66.7)	0(0)	0(0)
CYC, *n* (%)	4(21.1)	4(57.1)	1(33.3)	0(0)	0(0)
MMF, *n* (%)	3(15.8)	0(0)	0(0)	0(0)	0(0)
CsA, *n* (%)	2(10.5)	0(0)	0(0)	1(50.0)	0(0)
MTX, *n* (%)	1(5.3)	0(0)	0(0)	1(50.0)	0(0)

*AAV* ANCA-associated vasculitis, *CRP* C-reactive protein, *CsA* cyclosporine A, *CTD* connective tissue disease, *CYC* cyclophosphamide, *ESR* erythrocyte sedimentation rate, *GC* glucocorticoid, *IM* invasive mycoses, *IQR* interquartile range, *MMF* mycophenolate mofetil, *MTX* methotrexate, *PM/DM* polymyositis/dermatomyositis, *RA* rheumatoid arthritis, *SD* standard deviation, *SLE* systemic lupus erythematosus, *SSc* systemic sclerosis, *WBC* white blood cell

### Characteristics and laboratory data of IM in patients with CTD

The lung (30/32, 93.8%) was the most commonly involved. Two patients were diagnosed with fungal sinusitis (2/32, 6.3%) (Additional file 1: Figure S1 A-D). Pulmonary symptoms are presented in Table 2. *Aspergillus* spp. were the leading pathogens, causing 26 (81.3%) cases of infection. *Mucor* spp. and *Penicillium* spp. caused 3 (9.4%) and 2 (6.3%) infections, respectively. One (3.1%) patient was infected with both *Aspergillus* spp. and *Mucor* spp. Most of the causative fungi were identified in sputum culture (20/29, 69.0%), while a small proportion was found in BALF (3/10, 30.0%). *Aspergillus fumigates* was common (*n* = 10). Other causative agents included *Penicillium marneffei* (*n* = 2), *Mucor* (*n* = 2), *Aspergillus flavus* (*n* = 2), *Aspergillus niger* (*n* = 1), and *Rhizopus* (*n* = 1). Co-infection with mix fungi was found in 4 (12.5%) lupus patients and 1 (3.1%) AAV patient. Serum G test was performed in 23 patients and positive in 11 (47.8%). GM test was performed in 26 patients and positive in 9 (34.6%). The positive rate of the GM test was high in BALF (7/10, 70.0%). Six patients were diagnosed on the basis of biopsy, including four by bronchofiberscopy and two by nasal endoscopy (Additional file 1: Figure S1 G, H).

**Table 2:** Pulmonary manifestation of IM in patients with CTD

	Total (*n* = 30)
Clinical symptoms	
Fever, *n* (%)	20(66.7)
Cough, *n* (%)	24(80.0)
Sputum, *n* (%)	21(70.0)
Dyspnea, *n* (%)	8(26.7)
Hemoptysis, *n* (%)	6(20.0)
Chest distress, *n* (%)	3(10.0)
Chest pain, *n* (%)	2(6.7)
Radiographic manifestation	
Nodules, *n* (%)	14(46.7)
< 3 cm in diameter, *n* (%)	12(85.7)
> 3 cm in diameter, *n* (%)	2(14.3)
Single nodule, *n* (%)	6(42.9)
Multiple nodules, *n* (%)	8(57.1)
Cavitary lesions, *n* (%)	11(36.7)
Ground glass opacity, *n* (%)	8(26.7)
Halo sign, *n* (%)	3(10.0)
Location	
Unilateral involvement, *n* (%)	13(43.3)
Bilateral involvement, *n* (%)	17(56.7)

*CTD* connective tissue disease, *IM* invasive mycoses

### Pulmonary manifestation of IM in patients with CTD

Radiographic manifestation from pulmonary computed tomography (CT) scan is shown in Table 2. (Additional file 1: Figure S1E, F). Most of the nodules (85.7%) were small nodules with a diameter less than 3 cm. Nodules with blurred edges were found in 42.9% of the cases. A halo sign was reported in 3 (10.0%) cases. Nearly 28.6% of the nodules were located adjacent to the pleura. Bilateral involvement was common (56.7%). Patients with AAV had the highest rate of bilateral involvement (85.7%), followed by SLE (52.6%).

### Co-infection with other agents

Broad-spectrum antibiotics were given to 17 (53.1%) patients prior to IM diagnosis. Co-infection occurred in 11 (34.3%) patients, 10 of which were confirmed with etiological evidence. Co-infection occurred in 8 (42.1%) patients with SLE and 3 (42.9%) patients with AAV. Nearly 54.5% (6/11) of the co-infection occurred in the lung. The other five cases of infection involved multiple organs. Bacteria-associated infection accounted for 63.6% (7/11) of the cases. Tuberculosis occurred in 1 (9.1%) patient. Herpes zoster was diagnosed in 1 (9.1%) patient clinically. Co-infection with two pathogens occurred in 3 (27.3%) patients, 2 in SLE and 1 in AAV. Distributions of the infective sites and causal agents are shown in Additional file 2: Table S1.

### Treatment and outcomes

More than half (17/26, 65.4%) of the patients infected with *Aspergillus* spp. were treated with voriconazole. Other treatments included amphotericin B (3/26, 11.5%), caspofungin (2/26, 7.7%), itraconazole (2/26, 7.7%), and micafungin (1/26, 3.8%). Amphotericin B in combination with flucytosine was prescribed to four patients with mucormycosis. Patients infected with *P. marneffei* were given either caspofungin (1/2, 50.0%) or amphotericin B (1/2, 50.0%). Operation was performed in two patients diagnosed with fungal sinusitis and one patient with aspergilloma. Follow-up data was obtained from 29 patients and lost in 3. The median duration of follow-up was 9 months (IQR, 2.5, 15.0). Disease improved in 19 patients after anti-fungal treatment. Five patients (15.6%) were admitted to the intensive care unit (ICU). Ten patients died (10/32, 31.3%). Mortality was the highest in patients with AAV (3/7, 42.9%), followed by SLE (7/19, 36.8%). Infection with *P. marneffei* was the most fatal (mortality, 100%). Aspergillosis (mortality, 26.9%) and mucormycosis (mortality, 25.0%) ranked the second and the third. Among deceased patients with aspergillosis, 5 (71.4%) were infected with *A. fumigates*. Infection-induced respiratory failure was the primary cause (10/10, 100%). All the deaths occurred in patients with pulmonary IM (10/30, 33.3%). A comparison was performed between the survivors and non-survivors (Table 3). The incidence of leukopenia (4.5% vs 30.0%, *P* = 0.04) and lymphopenia (59.1% vs 100.0%, *P* = 0.02) was lower in the survivors. Elevated serum creatinine (27.3% vs 70.0%, *P* = 0.02) and co-infection (18.2% vs 70.0%, *P* = 0.004) were more frequent in non-survivors than survivors.

**Table 3:** Comparison between survivors and non-survivors with IM

Characteristics	Survivor (*n* = 22)	Non-survivor (*n* = 10)	*P* value
Demographic characteristics			
Sex, male to female	4:18	3:7	0.45
Age, years, mean ± SD	41.6 ± 15.6	44.2 ± 16.8	0.68
Diabetes mellitus, *n* (%)	2(9.1)	2(20.0)	0.39
Interstitial pneumonia, *n* (%)	4(18.2)	2(20.0)	0.90
Nephritis, *n* (%)	12(54.5)	7(70.0)	0.41
Laboratory data			
Leukopenia, *n* (%)	1(4.5)	3(30.0)	0.04*
Lymphopenia, *n* (%)	13(59.1)	10(100)	0.02*
Anemia, *n* (%)	12(54.5)	8(80.0)	0.17
Hypoalbuminemia, *n* (%)	14(63.6)	9(90.0)	0.12
Elevated serum creatinine, *n* (%)	6(27.3)	7(70.0)	0.02*
Co-infection, *n* (%)	4(18.2)	7(70.0)	0.004*
Co-infection with multiple agents, *n* (%)	1(4.5)	2(20.0)	0.22
Co-infection in multiple sites, *n* (%)	0(0)	5(50.0)	< 0.001*
Treatments prior to IM			
Median-to-high dose of GC, *n* (%)	7(31.8)	4(40.0)	0.65
CYC, *n* (%)	4(18.2)	5(50.0)	0.06
MMF, *n* (%)	1(4.5)	2(20.0)	0.16

*CYC* cyclophosphamide, *GC* glucocorticoid, *IM* invasive mycoses, *MMF* mycophenolate mofetil, *SD* standard deviation

^*^Significant

### Comparison between patients with or without IM

A total of 64 patients were selected as controls, including 38 patients with SLE, 14 patients with AAV, 6 patients with PM/DM, 4 patients with RA, and 2 patients with SSc. A comparison of the demographic and clinical characteristics is shown in Table 4. The prevalence of CTD-associated interstitial pneumonia (18.8% vs 3.1%, *P* = 0.01) and lymphopenia (71.9% vs 42.2%, *P* = 0.01) was more predominant in the mycoses group than in the control group. ESR (56 mm/h vs 27 mm/h, *P* = 0.01) and CRP levels (11.7 mg/l vs 2.9 mg/l, *P* = 0.002) were higher in patients with IM than in those without. A higher proportion of patients received median-to-high dose of GC (34.4% vs 9.4%, *P* < 0.001) in the mycoses group than those in the control group.

**Table 4:** Comparison between patients with or without IM

Characteristics	Case (*n* = 32)	Control (*n* = 64)	*P* value
Demographic characteristics			
Sex, male to female	7:25	15:49	0.86
Age, years, mean ± SD	42.4 ± 15.8	45.5 ± 17.5	0.44
Duration of CTD, months, median (IQR)	6.0(1.0,75.0)	24.0(7.5,87.0)	0.19
Diabetes mellitus, *n* (%)	4(12.5)	3(4.7)	0.17
Interstitial pneumonia, *n* (%)	6(18.8)	2(3.1)	0.01*
Nephritis, *n* (%)	19(59.4)	29(45.3)	0.19
Laboratory data			
Leukopenia, *n* (%)	4(12.5)	11(17.2)	0.55
Lymphopenia, *n* (%)	23(71.9)	27(42.2)	0.01*
Anemia, *n* (%)	20(62.5)	38(59.4)	0.77
Hypoalbuminemia, *n* (%)	23(71.9)	42(75.0)	0.75
Elevated serum creatinine, *n* (%)	13(40.6)	18(28.1)	0.22
IgM (g/l), median (IQR)	0.6(0.3,1.0)	0.8(0.5,1.3)	0.09
IgG (g/l), median (IQR)	14.1(10.3,17.3)	13.1(8.9,17.9)	0.77
ESR (mm/h),median (IQR)	56.0(40,66)	27.0(13,58)	0.01*
CRP (mg/l), median (IQR)	11.7(5.0,37.0)	2.9(0.9,14.3)	0.002*
Treatments prior to IM			
Median-to-high dose of GC, *n* (%)	11(34.4)	6(9.4)	< 0.001*
CYC, *n* (%)	9(28.1)	8(12.5)	0.06
MMF, *n* (%)	3(9.4)	8(12.5)	0.65
CsA, *n* (%)	3(9.4)	8(12.5)	0.65
MTX, *n* (%)	2(6.3)	6(9.4)	0.60

*CRP* C-reactive protein, *CsA* cyclosporine A, *CTD* connective tissue disease, *CYC* cyclophosphamide, *ESR* erythrocyte sedimentation rate, *GC* glucocorticoid, *IM* invasive mycoses, *IQR* interquartile range, *MMF* mycophenolate mofetil, *MTX* methotrexate, *SD* standard deviation

^*^Significant

Considering elevated ESR and CRP levels were non-specific to IM, only interstitial pneumonia, lymphopenia, and median-to-high dose of GC were included into the logistic regression analysis. Results of the logistic regression analysis are presented in Table 5. Lymphopenia (OR = 3.28, 95% CI 1.29–8.38, *P* = 0.01) and median-to-high dose of GC (OR = 3.40, 95% CI 1.04–11.13, *P* = 0.04) were associated with IM in patients with CTD.

**Table 5:** Factors associated with IM in patients with CTD

Characteristics	Univariate logistic regression			Multivariate logistic regression		
	Crude OR	95% CI	*P* value	Adjusted OR	95% CI	*P* value
Interstitial pneumonia	5.74	1.05–31.46	0.04*	–	–	**–**
Lymphopenia	3.50	1.40–8.76	0.01*	3.28	1.29–8.38	0.01*
Median-to-high dose of GC	3.78	1.21–11.83	0.02*	3.40	1.04–11.13	0.04*

*CI* confidence interval, *CTD* connective tissue disease, *GC* glucocorticoid, *IM* invasive mycoses, *OR* odds ratio

^*^Significant

### Subgroup analysis in patients with or without lymphopenia

Patients with CTD were divided into two groups according to the lymphocyte count. A subgroup analysis showed that patients with lymphopenia were more likely to develop co-infection than those without (50.0% vs 0%, *P* = 0.01). Infection-specific mortality was remarkably high (45.5% vs 0%, *P* = 0.01) in patients with lymphopenia (Table 6).

**Table 6:** Comparison between patients with or without lymphopenia

Characteristics	Normal (*n* = 10)	Lymphopenia (*n* = 22)	*P* value
Demographic characteristics			
Interstitial pneumonia, *n* (%)	1(10.0)	5(22.7)	0.39
Nephritis, *n* (%)	4(40.0)	15(68.2)	0.13
Bilateral lung involvement, *n* (%)	4(40.0)	15(68.2)	0.13
Co-infection, *n* (%)	0(0)	11(50.0)	0.01*
Laboratory data			
Anemia, *n* (%)	5(50.0)	15(68.2)	0.32
Hypoalbuminemia, *n* (%)	5(50.0)	18(81.8)	0.06
IgM (g/l), median (IQR)	0.9(0.4,1.1)	0.5(0.3,0.9)	0.24
IgG (g/l), median (IQR)	16.1(8.6,17.8)	14.1(12.1,16.7)	0.83
Treatments prior to IM			
Median-to-high dose of GC, *n* (%)	2(20.0)	9(40.9)	0.25
Use of immunosuppressant, *n* (%)	3(30.0)	12(54.5)	0.20
Mortality, *n* (%)	0(0)	10(45.5)	0.01*

*GC* glucocorticoid, *IQR* interquartile range

^*^Significant

## Discussion

In the present study, we found that patients with CTD were predisposed to IM. Patients with lymphopenia or treated with median-to-high dose of GC experienced high risk of IM and need fast and effective methods such as sputum culture or GM test in BALF to unveil the diagnosis. Patients with lymphopenia and impaired kidney function were extremely risky for fatal IM. Close monitoring is necessary in susceptible population to distinguish IM.

In our research, the incidence of IM was the highest in AAV (1.5%), compared to 0.5% in the SLE population [22]. Charles et al. reviewed 80 patients with AAV treated with rituximab and found three cases of aspergillosis (3.8%) [23]. Yoo et al. retrospectively reviewed 154 AAV patients and found 6 of them (3.9%) were infected with *Aspergillus* spp. [24]. Previous reports and ours suggested that patients with AAV were susceptible to IM. Therefore, clinicians should remain vigilant for IM, especially in AAV patients. Further research exploring the characteristics and determinants of fungal infection in AAV patients is necessary.

The lung is demonstrated to be the primary site of infection, with hidden sites including the sphenoid sinus. A prospective study on transplant recipients showed that most of the aspergillosis (78%) and zygomycosis (56%) was limited to the lung, and other infective sites also included the sinus (13%), cutaneous (13%) and disseminated sites (9%) [25]. Our research suggested that IM could occur in atypical sites in immunocompromised hosts, and careful examination is needed to uncover the potential fungal infection.

Although pathological evidence is the gold standard in diagnosing IM, the complex procedure usually delays prompt treatment. Alternatively, mycological culture and indirect test including the G test and GM test are recommended as valuable methods to facilitate IM diagnosis in immunocompromised hosts [19]. In our research, the positive rate of rapid diagnostic tests for IM was approximately 70.0%. Consistent with our results, GM antigen in BALF was suggested as a promising indicator in the diagnosis of mold infection [26, 27]. Our results further supported that sputum culture and GM test in BALF were rapid diagnostic methods for IM in patients with CTD.

High mortality of mycoses is widely recognized. One third of the patients (31.3%) with CTD died in our research. *P. marneffei* was the most fatal. These patients shared common clinical features of leukopenia, lymphopenia, and kidney impairment. Due to severe immunodeficiency, patients tended to develop co-infection with various pathogens. Practitioners may be able to influence the prognosis of IM if they remain mindful of the associated factors mentioned above and the increased risk of co-infection.

The toxicity of GC is well-recognized in immunocompromised hosts. GC use is also known to increase the risk of mix mold pulmonary infection in patients with hematological malignancies [28]. A high dose of GC was associated with the highest rate of serious infection in lupus patients [29]. Consistent with that, a median-to-high dose of GC was associated with IM and increased the risk 3.4-fold in our research. Lymphopenia was also significantly predominant in patients with IM than in those without. A cohort research has revealed that the risk of infection increased in parallel with the severity of lymphopenia in AAV patients [30]. The above findings suggested that lymphocyte count was a strong indicator for immunity surveillance. Accordingly, a subgroup analysis in the present study revealed that patients with lymphopenia were likely to develop mix infection and were associated with higher rates of mortality, and thus clinical vigilance is crucial to monitor the lymphocyte count during immunosuppressive treatment.

Our study has some drawbacks. The data used in this study was generated from one tertiary hospital from China. Thus, it could limit the generalizability of our findings to other populations. Furthermore, since this is a single-center research, only a small number of patients fulfilled the inclusion criteria. Conducting a larger study would strengthen and validate our result on the identified factors associated with IM. Additionally, biological agents are an important medication to treat CTD. However, no patient was given biologics prior to enrollment in our study. Biologics are mainly prescribed to patients with RA or SpA in China, but only a small number of patients with RA were included in this study. The impact of biologics on IM needs to be addressed in further research.

## Conclusions

This study demonstrated that IM are common comorbidities in patients with CTD, especially with AAV. Sputum culture and GM test in BALF, as fast and effective diagnostic methods, should be performed in suspected patients. Patients would benefit from close monitoring of the lymphocyte count, kidney function, and co-infection to reduce mortality.

### Additional files

Additional file 1: **Figure S1.** Radiographic and pathological manifestation of invasive mycoses (IM) in patients with connective tissue disease (CTD). (A) Computed tomography (CT) scan revealed soft tissue density (*red arrow*) filled in the sphenoid sinus. (B) T1-weighted magnetic resonance imaging (MRI) showed mildly increased signal intensity content (*red arrow*) in the sphenoid sinus. (C) Opacity with hypointensity (*red arrow*) was showed on the T2-weighted MRI. (D) Branched, septate hyphae of *Aspergillus* spp. (*asterisks*) were found in nasal sinus tissue obtained from nasal endoscopic biopsy (hematoxylin and eosin, HE staining, × 400). (E) A mass with blurred edge (*red arrow*) was located in the right lung. (F) An intracavitary nodule (*red arrow*) was shown on a background of pulmonary bullae in the left lung. (G) Fungal hyphae (*red arrows*) were observed in lung tissue obtained from bronchofibroscopy (HE staining, × 400). (H) Septate fungal hyphae (*red arrows*) were stained brown by periodic acid-silver methenamine (PASM) in lung tissue (× 400). (I) Periodic Acid-Schiff stain (PAS) revealed fungal hyphae (*red arrows*) in the lung tissue (× 400). (TIF 13028 kb)

Additional file 2: **Table S1.** Distributions of the infective sites and co-infective agents in patients with CTD. CTD, connective tissue disease; *MRSA*, methicillin-resistant *Staphylococcus aureus*. (DOCX 22 kb)

## Appendix

**Table 7:** The International Classification of Diseases-10 coding of CTD and invasive mycoses

Disease	ICD-10 coding
CTD	
MCTD	M35.105
PM/DM	M60.992, M60.896, M33.905, M33.202+, M33.201, M33.102+, M33.101
pSS	M35.001
RA	M06.991, M06.881, M06.001, M05.951, M05.309+, M05.306+, M05.303+, M05.302, M05.301, M05.001
SLE	M32.901, M32.155+
SpA	M45.X91, L40.501+
SSc	M34.902, M34.901, M34.807+, M34.806+, M34.801+
Vasculitis	D89.153, D69.001, N05.903, M31.003, M30.101, M30.001, L95.902, L95.901, I77.851, I77.607, I77.605, I77.502, I77.601, M31.601, M31.505
Invasive mycoses	
Aspergillosis	B44.051+, B44.101+, B44.102+, B44.103+, B44.151, B44.751, B44.752, B44.801, B44.901
Coccidioidomycosis	B38.052+, B38.151+, B38.201+, B38.451+, B38.751, B38.752, B38.901
Fungal disease	B49.X51
Mucormycosis	B46.001+, B46.151+, B46.251+, B46.451, B46.452, B46.501
Mycosis	B48.751, B49.XO1, B49.XO2, B49.XO4+, B49.XO5, B49.XO6, B49.XO7+, B49.XO9+, B49.X10+, B49.X11, B49.X12+, B49.X13+, B49.X14+, B49.X15, B49.X16+
Penicilliosis	B48.451
Zygomycosis	B46.952

*ICD-10* International Classification of Diseases-10, *CTD* connective tissue disease, *MCTD* mixed connective tissue disease, *PM/DM* polymyositis/dermatomyositis, *pSS* primary Sjogren’s syndrome, *RA* rheumatoid arthritis, *SLE* systemic lupus erythematosus, *SpA* spondyloarthrosis, *SSc* systemic sclerosis

## Abbreviations

AAV: ANCA-associated vasculitis
BALF: Bronchoalveolar lavage fluid
CI: Confidence interval
CRP: C-reactive protein
CsA: Cyclosporine A
CT: Computed tomography
CTD: Connective tissue disease
CYC: Cyclophosphamide
ESR: Erythrocyte sedimentation rate
GC: Glucocorticoid
GM: Serum galactomannan
HE: Hematoxylin and eosin
ICU: Intensive care unit
IFD: Invasive fungal disease
IPA: Invasive pulmonary aspergillosis
MCTD: Mixed connective tissue disease
MMF: Mycophenolate mofetil
MRI: Magnetic resonance imaging
MTX: Methotrexate
OR: Odds ratio
PAS: Periodic Acid-Schiff stain
PASM: Periodic acid-silver methenamine
PM/DM: Polymyositis/dermatomyositis
RA: Rheumatoid arthritis
SLE: Systemic lupus erythematosus
SpA: Spondyloarthrosis
SSc: Systemic sclerosis
