# Supplementary figures and images for: Invasive mycoses in patients with connective tissue disease from Southern China: clinical features and associated factors
Source: Arthritis Res Ther. 2019 Mar 11;21:71. doi: 10.1186/s13075-019-1851-9 (PMC6416859; doi:10.1186/s13075-019-1851-9)

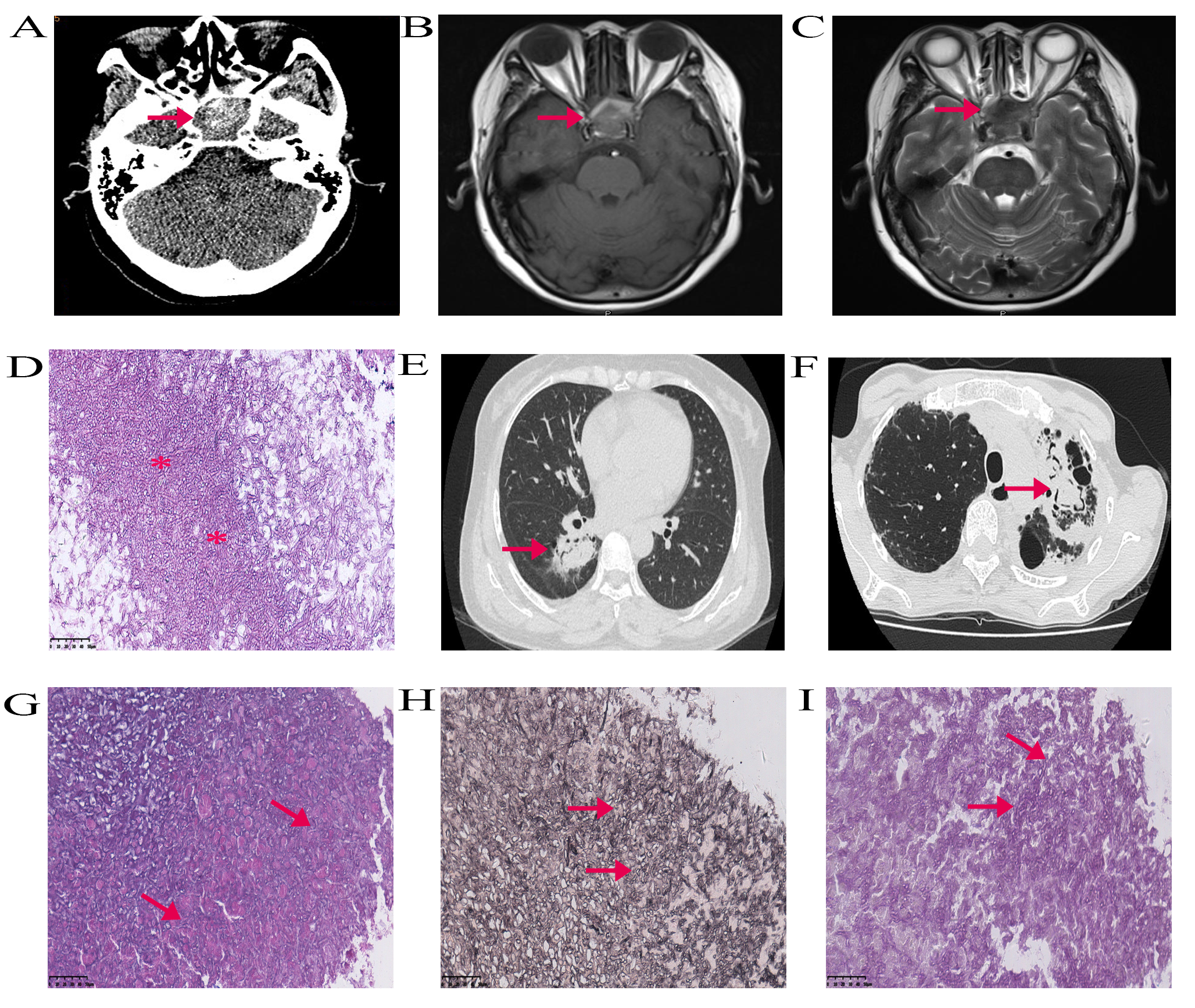

Supplement: Supplementary file 1 — Figure S1. Radiographic and pathological manifestation of invasive mycoses (IM) in patients with connective tissue disease (CTD). (A) Computed tomography (CT) scan revealed soft tissue density (red arrow) filled in the sphenoid sinus. (B) T1-weighted magnetic resonance imaging (MRI) showed mildly increased signal intensity content (red arrow) in the sphenoid sinus. (C) Opacity with hypointensity (red arrow) was showed on the T2-weighted MRI. (D) Branched, septate hyphae of Aspergillus spp. (asterisks) were found in nasal sinus tissue obtained from nasal endoscopic biopsy (hematoxylin and eosin, HE staining, × 400). (E) A mass with blurred edge (red arrow) was located in the right lung. (F) An intracavitary nodule (red arrow) was shown on a background of pulmonary bullae in the left lung. (G) Fungal hyphae (red arrows) were observed in lung tissue obtained from bronchofibroscopy (HE staining, × 400). (H) Septate fungal hyphae (red arrows) were stained brown by periodic acid-silver methenamine (PASM) in lung tissue (× 400). (I) Periodic Acid-Schiff stain (PAS) revealed fungal hyphae (red arrows) in the lung tissue (× 400). (TIF 13028 kb) [file 13075_2019_1851_MOESM1_ESM.tif]
