# Supplementary material for: Invasive mycoses in patients with connective tissue disease from Southern China: clinical features and associated factors
Source: Arthritis Res Ther. 2019 Mar 11;21:71. doi: 10.1186/s13075-019-1851-9 (PMC6416859; doi:10.1186/s13075-019-1851-9)
Supplement: Supplementary file 2 — Table S1. Distributions of the infective sites and co-infective agents in patients with CTD. CTD, connective tissue disease; MRSA, methicillin-resistant Staphylococcus aureus. (DOCX 22 kb) [file 13075_2019_1851_MOESM2_ESM.docx]

Additional file 2: Table S1

| **Characteristics of co-infection** | |
| --- | --- |
| **Agents** |  |
| Bacteria |  |
| *Gram-negative* | *P. Aeruginosa* (5), *K. Pneumoniae* (3), *A. Baumanii* (2), *S. Maltophilia* (1) *E. Coli* (1), *A. Caviae* (1), *B. Cepacia* (1), *F. Indologenes* (1), *C. Freundii* (1) |
| *Gram-positive* | *MRSA* (1), *Star nocardia* (1), *Enterococcus faecium* (1) |
| Mycobacterium | *M. tuberculosis* (2) |
| Virus | Herpes Zoster (1) |
| Superficial fungi | *Candida glabrata* (1) |
| **Infective sites** | Lung (6), Lung+Blood stream (2), Lung+Urinary tract (1), Lung+Brain (1), Lung+Blood stream+Urinary tract (1) |
